# Choriocarcinoma in a 73-year-old woman: a case report and review of the literature

**DOI:** 10.1186/1752-1947-4-379

**Published:** 2010-11-25

**Authors:** Nisarg R Desai, Shilpi Gupta, Rabih Said, Priyal Desai, Qun Dai

**Affiliations:** 1 Department of Medicine, Staten Island University Hospital, Staten Island, New York, USA; 2 Department of Hematology and Oncology, Staten Island University Hospital, Staten Island, New York, USA; 3 New Civil Hospital, Surat, India

## Abstract

**Introduction:**

Choriocarcinoma is a highly malignant tumor of trophoblastic origin. Most cases present within one year of the antecedent pregnancy (molar or non-molar). However, very rarely, choriocarcinoma can develop from germ cells or from dedifferentiation of endometrial carcinoma into choriocarcinoma. This article concerns a case of choriocarcinoma developing 38 years after the patient's last pregnancy and 23 years after menopause.

**Case presentation:**

A 73-year-old African-American woman presented with a three-week history of vaginal bleeding. A vaginal mass was seen on pelvic examination. Ultrasonography showed a thickened complex endometrial echo. Her β-human chorionic gonadotrophin level was found to be elevated (2,704,040 mIU/mL). Vaginal and uterine biopsies were suggestive of choriocarcinoma. Immunohistochemistry tests were positive for β-human chorionic gonadotrophin as well as cytokeratin and negative for octamer binding transcription factor 3/4 and α-fetoprotein, supporting the diagnosis of choriocarcinoma. A combination of etoposide, methotrexate, and dactinomycin, followed by cyclophosphamide and vincristine (the so-called EMA/CO regimen) was initiated. After seven cycles of chemotherapy, her β-human chorionic gonadotrophin level dropped below 5 mIU/mL. Our patient is being followed up at our oncology institute.

**Conclusions:**

We report an extremely rare case of choriocarcinoma arising 23 years after menopause. A postmenopausal woman presenting with vaginal bleed from a mass and β-human chorionic gonadotrophin elevation should be evaluated by immunohistochemical analysis to rule out the possibilities of a germ cell origin of the tumor or dedifferentiation of an epithelial tumor. Absence of octamer binding transcription factor 3/4, α-fetoprotein and CD-30 staining helps in exclusion of most germ cell tumors. DNA polymorphism studies can be used to differentiate between gestational and non-gestational tumor origin. These require fresh tissue samples and are time consuming. Finally, the effective first-line therapy for β-human chorionic gonadotrophin-producing high-risk gestational as well as non-gestational trophoblastic tumors is combination chemotherapy (the EMA/CO regimen). Therefore, treatment should be commenced when a potential diagnosis of metastatic trophoblastic tumor is being considered.

## Introduction

Choriocarcinoma is a highly malignant trophoblastic tumor composed of two types of cells, syncytiotrophoblasts and cytotrophoblasts. The syncytiotrophoblast is the differentiated hormone secreting component [1,2]. Most cases of choriocarcinoma are intra-uterine and of gestational origin. Extrauterine gestational choriocarcinomas may also arise at a site of ectopic pregnancy. The non-gestational choriocarcinomas are believed to develop from pluripotent germ cells, most commonly arising in the gonads. Finally, various poorly differentiated carcinomas may show focal area of choriocarcinomatous differentiation [1,3]. Gestational choriocarcinoma is a rare complication of pregnancy (incidence of one in 20,000 to one in 25,000 in western countries) and usually arises from a prior molar pregnancy or rarely a non-molar gestation, within one year of the antecedent pregnancy [4]. Choriocarcinoma in postmenopausal woman is very rare, however a few cases of choriocarcinoma developing after a long latent period from last pregnancy have been reported [4-7]. Here, we describe a case of choriocarcinoma in a 73-year-old woman developing 38 years after her last pregnancy and 23 years after her last menstrual period.

## Case presentation

**Figure 1:**
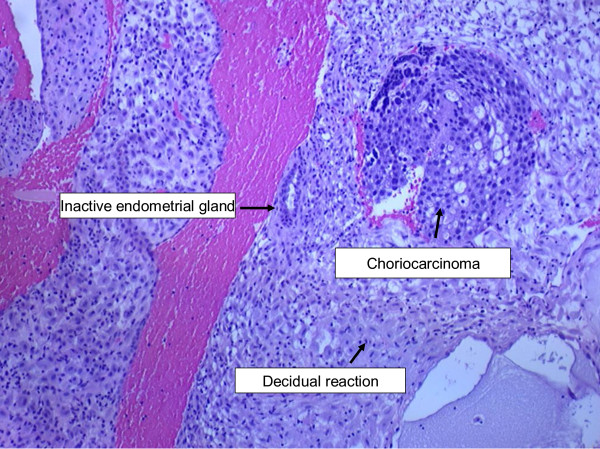
**Endometrial biopsy showing choriocarcinoma with decidual reaction**.

**Figure 2:**
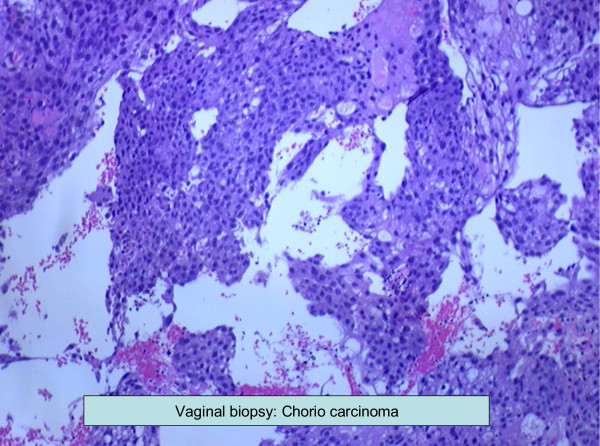
**Vaginal biopsy showing choriocarcinoma**.

**Figure 3:**
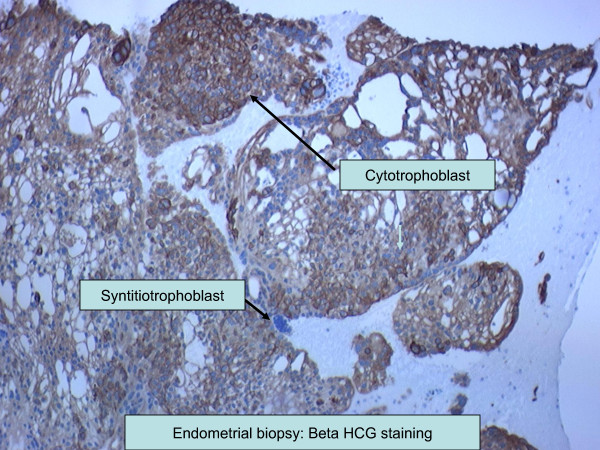
**Immunohistochemistry analysis of endometrial biopsy specimen**. Cytotrophoblasts and syncytiotrophoblasts were stained with β-human chorionic gonadotrophin (β-hCG) antibody.

A 73-year-old African-American woman, gravida 4 para 4, presented with a three-week history of postmenopausal vaginal bleeding, with associated suprapubic pain and urinary retention for the past two days. A pelvic exam revealed a 5 cm fungating left vaginal wall mass extending to the bladder trigone, and a closed cervix. There was no cervical motion tenderness and no palpable adnexal mass. Our patient had suprapubic tenderness with no palpable mass in her abdomen. All other examinations were unremarkable. Pelvic and transvaginal sonograms showed a thickened complex endometrial echo (2.4 cm) and her uterus measured 9.7×6.2×5.4 cm. Her ovaries were normal in size (2.5×1.8×1.5 cm). Computed tomography (CT) scans of the chest, abdomen and pelvis showed a heterogeneous vagina and two hepatic masses measuring 7.7 cm and 3.4 cm, respectively. A CT scan of her brain with contrast and a bone scan did not show any evidence of metastasis. Two biopsies were taken from the endometrial and vaginal wall masses. Grossly, the endometrial biopsy consisted of multiple fragments of blood clots and grayish tissue, 3.9 cm in aggregate. The vaginal wall biopsy consisted of multiple fragments of brown-red, soft and firm tissue, measuring 3.3 cm in aggregate. Histological examination was suggestive of choriocarcinoma. The non-lesional endometrium showed decidualization (Figures 1 and 2). On immunohistochemistry tumor cells appeared positive for β-human chorionic gonadotrophin (β-hCG) (Figure 3) and cytokeratins (AE-1, AE-2) and negative for octamer binding transcription factor (OCT)-3/4, α-fetoprotein (AFP) and CD-30. There was no histological evidence of any other type of malignancy (no germ cell component, no endometrial carcinoma). Percutaneous CT-directed core needle biopsy of the larger liver lesion demonstrated extensive necrosis with atypical cells suggestive of malignancy.

Our patient's β-hCG level was 2,704,040 mIU/mL. Cancer antigen 125 (CA-125) and AFP levels were normal. Chemotherapy with etoposide, methotrexate and dactinomycin, followed by cyclophosphamide and vincristine (the so-called EMA/CO regimen) was initiated. Her β-hCG level at follow-up (after the first cycle of chemotherapy) was 646 mIU/mL. Her vaginal bleeding and urinary symptoms resolved after the first cycle of chemotherapy. After seven cycles of EMA/CO chemotherapy her β-hCG level dropped below 5 mIU/mL. A repeat CT scan (after four months) showed a normal-appearing uterus and a decrease in size of the metastatic liver masses (4.5 cm and 1.7 cm). Our patient is now clinically asymptomatic and is on regular follow-up at our cancer center. Her β-hCG level stayed below 5 mIU/mL at one year of follow-up.

Our patient's obstetric history was significant for four normal vaginal deliveries (the last at 35 years of age) and menopause at 50 years of age. She denied any episode of postmenopausal bleeding before this presentation.

## Discussion

Choriocarcinomas can be divided into two types: gestational and non-gestational. Gestational choriocarcinomas mostly occur in woman of reproductive age, usually within one year following a molar or non-molar pregnancy. Non-gestational choriocarcinomas can arise from germ cell or trophoblastic differentiation within endometrial carcinomas. Extraovarian germ cell tumors, including choriocarcinomas may arise from germ cells that failed to complete their migration to the gonads [8]. However, germ cell choriocarcinomas arising from the female genital tract in postmenopausal woman with normal ovaries on CT scan and sonography are extremely rare [3,9].

When choriocarcinoma occurs in postmenopausal woman, it is difficult to rule out the possibility of trophoblastic differentiation within an endometrial carcinoma. Choriocarcinoma has been reported in association with endometrial carcinoma as well as liver, lung and urinary bladder carcinomas [10]. These types of choriocarcinomas can be diagnosed based on histology (that is, coexisting malignant cells other than choriocarcinoma cells). Khuu *et al*. reported a case of uterine carcinosarcoma with choriocarcinomatous dedifferentiation in a 71-year-old woman [10]. In that case, histology results suggested choriocarcinoma intermixed with adenocarcinoma and stromal sarcoma. While, in our patient, there was no evidence of endometrial adenocarcinoma. The non-lesional endometrium showed decidualization. These findings would rule out dedifferentiation within an endometrial carcinoma. A report from Chumworathayi *et al*. described cervical choriocarcinoma with metaplastic transformation from squamous cells [11]. The authors discussed the possibility of *in situ *squamous cell carcinoma, which may not be initially diagnosed on small tissue biopsy. In our patient, an endometrial biopsy showed malignant syncytiotrophoblasts and cytotrophoblasts associated with inactive, non-malignant endometrial glands and decidual reaction in normal-appearing endometrium (Figure 1).

Immunohistochemistry analysis is useful in differential diagnosis of choriocarcinoma. Strong diffuse β-hCG immunoreactivity confirms the diagnosis of choriocarcinoma. OCT-3/4, CD-30 and AFP are markers of various germ cell tumors [12]. OCT-3/4 is a transcription factor, expressed in undifferentiated pluripotent cells including germ cells. CD-30 is a member of the tumor necrosis factor superfamily of cytokine receptors. Positive staining for CD-30 has been used for diagnosis of embryonal carcinoma. OCT-3/4 and CD-30 can be used in combination to establish the germ cell origin of any metastatic tumor. Negative staining for both markers helps in ruling out a germ cell origin of such tumors [12-14]. AE1/AE3 is a combination of two pancytokeratin antibodies, AE1 and AE3. AE1/AE3 staining is usually positive in choriocarcinomas as cytokeratin is expressed on trophoblastic cells (trophoblastic cells are derived from epithelial cells) [13,15]. Various serum tumor markers (β-hCG, AFP and CA-125) are also useful in the differential diagnosis of choriocarcinoma. It is well known that elevated AFP and CA-125 levels are seen in non-seminomatous germ cell tumors and ovarian carcinomas, respectively [16]. As discussed above, in our patient immunohistochemistry analysis was positive for β-hCG (Figure 1) and cytokeratins (AE-1/AE-3 antibodies), while staining of OCT-3/4, CD-30 and AFP was negative. Therefore, negative staining with OCT-3/4, CD-30 and AFP antibodies as well as normal AFP and CA-125 levels suggested a gestational origin of the tumor.

Fisher *et al*. demonstrated DNA polymorphism studies are the most specific to confirm a gestational origin of tumor [3]. These studies compare microsatellite polymorphism between the patient, tumor and partner's DNA (if available) by examination of restriction fragment length polymorphisms (RFLPs) using locus specific microsatellites. Genetic studies are useful when the patient's history and pathological review are insufficient for diagnosis. However, they are time consuming and do not always give conclusive results.

Based on American Joint Committee on Cancer (AJCC) staging guidelines for gestational trophoblastic tumors (GTT), our patient had stage IVB cancer (considering high β-hCG and clinical liver metastasis) [17]. Treatment guidelines for choriocarcinomas in postmenopausal woman are not well defined. However, previous studies have suggested that an effective first-line therapy for high-risk gestational trophoblastic tumor is the combination of etoposide, methotrexate, and dactinomycin, followed by cyclophosphamide and vincristine (the EMA/CO regimen) [18]. However, full genetic analysis from tumor biopsies and patient DNA is time consuming and does not always yield conclusive results [3]. Therefore, based upon histology, serum tumor markers and immunochemistry analysis, we initiated treatment with EMA-CO. Our patient responded well to this regimen as seen clinically (resolution of vaginal bleeding), as well as radiologically (a normal-appearing uterus and decrease in size of metastatic liver lesions). Her response to chemotherapy was confirmed by a decrease in β-hCG level. The chemotherapy regimen was repeated every two weeks for five cycles. At the end of five cycles, our patient's β-hCG level plateaued around 10 mIU/mL. We repeated this regimen for two more cycles until remission (normalization of β-hCG) was achieved.

The molecular mechanism behind the long latent period between development of a choriocarcinoma and last pregnancy has not been described. In our patient, it is theoretically possible that she became pregnant after her last known gestation (before 38 years) but without clinical symptoms. However, our patient considers this to be unlikely. Even if we consider the possibilities of asymptomatic gestation developing in choriocarcinoma, our patient has an established menopause of 23 years. To date, there are very few case reports in the global literature of gestational diseases in postmenopausal women. Reports of choriocarcinoma are even more rare [1,3,4,9-11,19-21]. Tsukamoto *et al*. reported three postmenopausal patients with choriocarcinoma, with the periods between the last pregnancy and development of tumor being 11, 15 and 18 years [22]. O'Neill *et al*. and Okamoto *et al*. reported choriocarcinoma 22 and 23 years after last pregnancy, respectively [4,5].

## Conclusions

To the best of our knowledge, this is the first case of choriocarcinoma after a latent period of 38 years after last pregnancy and 23 years after menopause [3-5,9,19,20]. Germ cell choriocarcinoma confirmed by DNA analysis is extremely rare and has previously only been reported in women of child bearing age [3,23]. In our patient's case, we do not rule out the possibility of a non-gestational choriocarcinoma, but the response to chemotherapy with histology, immunohistochemistry and serum tumor markers suggested a gestational origin. The prognosis of germ cell choriocarcinoma is extremely poor despite chemotherapy and surgery. Therefore, we encourage not delaying patient management while awaiting DNA analysis results. Treatment with a combination chemotherapy regimen such as EMA/CO should be initiated immediately after establishing a diagnosis of choriocarcinoma with the help of histology, tumor markers and immunohistochemistry.

## Consent

Written informed consent was obtained from the patient for publication of this case report and any accompanying images. A copy of the written consent is available for review by the Editor-in-Chief of this journal.

## Competing interests

The authors declare that they have no competing interests.

## Authors' contributions

NRD designed the article, performed the literature search and wrote approximately 70% of the article. NRD also helped in formatting article and had a role in submission. SG, RS, PD, QD helped in designing article, and wrote 30% of the article. They also helped in editing the article. All authors read and approved the final manuscript.
